# A Systematic Review on Bleeding Risk Scores’ Accuracy after Percutaneous Coronary Interventions in Acute and Elective Settings

**DOI:** 10.3390/healthcare9020148

**Published:** 2021-02-02

**Authors:** Crischentian Brinza, Alexandru Burlacu, Grigore Tinica, Adrian Covic, Liviu Macovei

**Affiliations:** 1 Institute of Cardiovascular Diseases “Prof. Dr. George I.M. Georgescu”, 700503 Iasi, Romania; crischentian-branza@email.umfiasi.ro (C.B.); grigore.tinica@umfiasi.ro (G.T.); liviu.macovei@umfiasi.ro (L.M.); 2 Faculty of Medicine, University of Medicine and Pharmacy “Grigore T Popa”, 700115 Iasi, Romania; adrian.covic@umfiasi.ro; 3 Medical Sciences Academy, 030167 Bucharest, Romania; 4 Nephrology Clinic, Dialysis, and Renal Transplant Center—‘C.I. Parhon’ University Hospital, 700503 Iasi, Romania

**Keywords:** bleeding risk scores, percutaneous coronary intervention, bleeding events, dual antiplatelet therapy, systematic review

## Abstract

Dual antiplatelet therapy (DAT) is recommended for all patients undergoing percutaneous coronary intervention (PCI), as it significantly reduces the ischemic risk at the cost of increasing the incidence of bleeding events. Several clinical predictive models were developed to better stratify the bleeding risk associated with DAT. This systematic review aims to perform a literature survey of both standard and emerging bleeding risk scores and report their performance on predicting hemorrhagic events, especially in the era of second-generation drug-eluting stents and more potent P2Y12 inhibitors. We searched PubMed, ScienceDirect, and Cochrane databases for full-text studies that developed or validated bleeding risk scores in adult patients undergoing PCI with subsequent DAT. The risk of bias for each study was assessed using the prediction model risk of bias assessment tool (PROBAST). Eighteen studies were included in the present systematic review. Bleeding risk scores showed a modest to good discriminatory power with c-statistic ranging from 0.49 (95% CI, 0.45–0.53) to 0.82 (95% CI, 0.80–0.85). Clinical models that predict in-hospital bleeding events had a relatively good predictive performance, with c-statistic ranging from 0.70 (95% CI, 0.67–0.72) to 0.80 (95% CI, 0.73–0.87), depending on the risk scores and major hemorrhagic event definition used. The knowledge and utilization of the current bleeding risk scores in appropriate clinical contexts could improve the prediction of bleeding events.

## 1. Introduction

Dual antiplatelet therapy (DAT) has been a vital need once percutaneous coronary interventions (PCI) became available and different types of coronary stents were developed (bare-metal stents, BMS, and three generations of drug-eluting stents, DES). The risk of acute, subacute, late, or very late stent thrombosis represents a vital concern that is significantly reduced by implementing DAT. The ischemic risk was improved after the discovery and clinical use of new potent antiplatelet agents, P2Y12 antagonists, and third-generation drug-eluting stents, which carry a lower risk of thrombosis even after two years, compared to BMS [1,2,3,4]. DAT with Aspirin and a P2Y12 antagonist after percutaneous coronary angioplasty with BMS or DES is the standard of care recommended by all guidelines.

However, apart from reducing the incidence of ischemic events, DAT also increases the bleeding risk. As these patients are on the edge between ischemic and hemorrhagic events, careful examination of the patient risk profile is recommended. Nevertheless, patients may oftentimes exhibit a high risk of ischemic and bleeding events simultaneously, leading to a clinical and therapeutic dilemma. The duration of DAT is another issue frequently encountered in clinical practice, since the bleeding risk may exceed the ischemic risk after a certain time interval of DAT administration. This blurred line between switching to a single antiplatelet agent after DAT discontinuation and maintaining DAT is of considerable debate in clinical studies. Many bleeding risk scores were developed to assist in choosing the adequate therapeutic regimen and its duration, some of the scores being externally validated [5,6,7,8] (Table S1).

The American College of Cardiology (ACC)/American Heart Association (AHA) guidelines focusing on the duration of DAT were published before the development and validation of the Predicting Bleeding Complication in Patients Undergoing Stent Implantation and Subsequent Dual Antiplatelet Therapy (PRECISE-DAPT) risk score. Thus, the DAPT score was the only one available at that moment [9,10]. The position of the ACC/AHA Task Force is that the DAPT score may be useful in tailoring DAT duration after one year, giving more importance to the clinical judgment and careful evaluation of hemorrhagic and ischemic risk factors.

The European Society of Cardiology (ESC) and the European Association for Cardio-Thoracic Surgery (EACTS) guidelines that focused on the DAT topic advocated for a personalized approach regarding bleeding and ischemic risks in the detriment of the generalized strategy. The task force recommends (class IIB, level of evidence A) the use of dedicated scores to evaluate a patient’s risk profile to establish the duration of DAT in different clinical settings. PRECISE-DAPT and DAPT are the recommended risk scores by the Task Force [11]. According to the Task Force, patients presenting with CCS treated with DES or BMS can benefit from standard or long-term DAT (6 months or more) if they are not at increased bleeding risk (PRECISE-DAPT < 25) or short DAT (1–3 months) if they are at high risk of bleeding (PRECISE-DAPT ≥ 25). Patients with ACS who underwent PCI with DES or BMS with a high bleeding risk (PRECISE-DAPT ≥ 25) could be treated with a short DAT regimen (6 months), and those with non-high bleeding risk (PRECISE-DAPT < 25) could benefit from a longer DAT duration (12 months or more). After 12 months of DAT in patients without hemorrhagic events, DAPT score can be further used to tailor DAT duration.

However, the lack of a perfect risk score with a substantial predictive value led to further research and development of clinical models in addition to PRECISE-DAPT and DAPT scores: Patterns of Non-Adherence to Anti-Platelet Regimen in Stented Patients (PARIS), Can Rapid risk stratification of Unstable angina patients Suppress ADverse outcomes with Early implementation of the ACC/AHA guidelines (CRUSADE), The Academic Research Consortium for high bleeding risk (ARC-HBR), Acute Catheterization and Urgent Intervention Triage strategY—Harmonizing Outcomes with Revascularization and Stents in Acute Myocardial Infarction (ACUITY-HORIZONS), Bleeding complications in a Multicenter registry of patients discharged with a diagnosis of Acute Coronary Syndrome (BleeMACS), ACTION, Mehran. 

In the last few years, new clinical models were developed and were validated in various clinical scenarios to better predict hemorrhagic events in patients treated with DAT following PCI. The objective of this updated systematic review was to search the literature for classic and newer bleeding risk scores and their predictive performance, especially in the era of second-generation DES and more potent P2Y12 inhibitors.

## 2. Materials and Methods

The systematic review was conducted according to Preferred Reporting Items for Systematic Review and Meta-Analysis (PRISMA) checklist [12].

### 2.1. Data Sources

The search was performed in two steps (March 2020 and November 2020) in PubMed, ScienceDirect and Cochrane databases, using the following terms: “PRECISE-DAPT”, “DAPT”, “PARIS”, “ACUITY”, “CRUSADE”, “ARC-HBR”, “BleeMACS”, “bleeding risk score”, “dual antiplatelet therapy”, “stratifying bleeding risk”, “percutaneous coronary intervention”, “major bleeding”, and “predictive value”. The search was restricted to trials involving humans and published in English.

### 2.2. Study Selection

Studies were eligible if they reported original data regarding the discriminatory power of the risk scores used to predict bleeding events associated with DAT, as the primary or secondary outcome in adult patients undergoing PCI for acute or chronic coronary syndromes (ACS and CCS, respectively). Several exclusion criteria were established: studies available only in abstract, the outcome of interest was not reported, meta-analyses, bleeding events were not defined as minor or major according to The Bleeding Academic Research Consortium (BARC), The Thrombolysis in Myocardial Infarction (TIMI), Global Use of Strategies to Open Occluded Coronary Arteries (GUSTO) or other definitions based on local expertise. In addition, studies that focused exclusively on triple antithrombotic therapy were excluded.

### 2.3. Data Extraction

The following data were extracted from each study: design, the number of patients included, the clinical setting, the evaluated bleeding risk score and its discriminatory power, the criteria used in grading bleeding events, the proportion of major bleeding events, and the time interval. Disagreements were resolved by consensus. When available, data are presented as percentages, ranges of variation, mean or median values, c-statistic/Area Under the Receiver Operating Characteristics (AUC/AUROC), and confidence intervals (CIs). In addition, when possible, the sensitivity, specificity, and accuracy of the bleeding risk scores are reported.

### 2.4. Outcomes

We assessed the predictive performance of different bleeding risk scores reported in clinical trials that included patients with DAT after PCI in an in-hospital setting or following discharge after hemorrhagic events.

### 2.5. Risk of Bias

The risk of bias and applicability concern for each study were appraised using the Prediction model Risk Of Bias Assessment Tool (PROBAST), which was designed for diagnostic and prognostic clinical models in the setting of development or validation [13]. PROBAST checklist comprises four domains (participants, predictors, outcome, analysis) with different signaling questions, which evaluate the overall risk of bias and applicability.

## 3. Results

**Figure 1:**
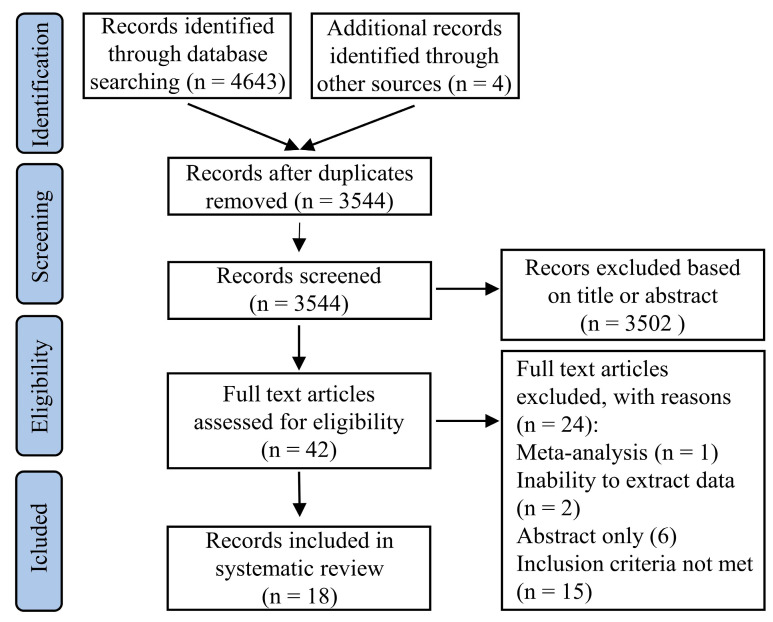
Flow diagram of the studies selection for inclusion in the systematic review.

Our search in databases identified 4647 citations. After screening for duplicates and reviewing the titles and abstracts, 4605 citations were excluded. Of the remaining 42 studies, one meta-analysis and six abstracts were excluded. An additional 17 studies were excluded because inclusion criteria were not met or due to the inability to extract data, leaving 18 trials included (Figure 1).

Each study and population characteristics are summarized in Table 1. 

Of 18 studies, eight were single-center: South Korea [14], Spain [15,16,17], Switzerland [18], USA [19], China [20], Sweden [21], and ten were performed in multiple centers [22,23,24,25,26,27,28,29,30,31]. Two studies evaluated the risk scores for predicting in-hospital bleeding events [16,17], while the others assessed the clinical models for predicting hemorrhagic events associated with long-term DAT (≤1 year—eight studies and >1 year—eight studies).

The most often used bleeding definition was provided by BARC, which was followed by TIMI and GUSTO. Two studies defined major hemorrhagic events based on local expertise [16,26]. Six studies [15,16,17,25,26,29] evaluated the performance of bleeding risk scores in the context of ACS with PCI and subsequent DAT, including two studies focused on ST-elevation myocardial infarction (STEMI) [16,17].

Essential differences were reported regarding the P2Y12 antagonist used as part of DAT. In this regard, Clopidogrel was the most investigated drug in studies. Seven clinical trials used more potent P2Y12 antagonists (Ticagrelor or Prasugrel) to a greater extent [15,18,19,23,25,29,30]. There were differences in the type of stent used (DES or BMS) and the proportion of patients needing oral anticoagulant therapy for the long-term.

The comparative predictive performances described as the discriminatory power (c-statistic) of the bleeding risk scores, as reported in each study, are illustrated in Table S2. Overall, the bleeding risk scores demonstrated a modest to good discriminatory power, with c-statistic ranging from 0.49 (95% CI, 0.45–0.53) to 0.82 (95% CI, 0.80–0.85). 

The highest c-statistic of 0.82 was recorded by Choi et al. [14], in the case of the PRECISE-DAPT risk score when bleeding events were defined by GUSTO criteria. Of note, in the derivation cohort of PRECISE-DAPT bleeding risk score, and validation cohorts—PLATelet inhibition and patient Outcomes (PLATO) trial and BernPCI registry [22], the c-statistic was significantly lower (0.73, 0.70, and 0.66 respectively). The PRECISE-DAPT score was also validated by Choi et al. [29] in patients with ACS with a similar prediction power, and by Kawashima et al. [30], who reported a lower c-statistic value (0.648). Gragnano et al. [31] assessed the classic PRECISE-DAPT score’s performance and the four-item PRECISE-DAPT score performance, which was recently developed. The c-statistic was similar at one year and two years, ranging between 0.60 and 0.66. 

Two studies [23,24] developed and validated DAPT and PARIS risk scores for predicting bleeding events in patients with long-term DAT, beyond one year. DAPT and PARIS bleeding risk scores showed a modest discrimination power in derivation (c-statistic 0.68 and 0.75 respectively) and validation cohorts (c-statistic 0.64 for both scores). However, the population covered was different because the PARIS study included only patients who underwent PCI with DES. The DAPT score was also evaluated by Ueda et al. [21], in a large cohort of patients—Swedish Web-system for Enhancement and Development of Evidence-based care in Heart disease Evaluated According to Recommended Therapies (SWEDEHEART) registry, but the c-statistic was unsatisfactory (0.49). However, Song et al. [20] reported an excellent prediction power regarding DAPT score (0.71), but there were a few hemorrhagic events.

Abu-Assi et al. [15] and Bianco et al. [25] comparatively appraised the predictive performance of PRECISE-DAPT and PARIS risk scores in case of patients with ACS treated with PCI. In the former study, the authors reported a similar discrimination power for both risk scores (c-statistic 0.73), but the discrimination power was lower in the latter study, especially in the PARIS score (c-statistic 0.653 for PRECISE-DAPT score and 0.593 for PARIS score).

ARC-HBR, a newer bleeding risk score, was validated by Ueki et al. [18] and Cao et al. [19] in patients treated with DAT following PCI with stenting. This bleeding risk score had a similar discrimination power (respectively, c-statistic 0.67 and 0.68). 

BleeMACS is another bleeding risk score developed and validated internally and externally by Raposeiras-Roubin et al. [26,32]. In large derivation and validation cohorts, the authors reported a modest predictive power for bleeding events (c-statistic 0.71 in derivation cohort). This score was externally validated in SWEDE-HEART registry, with a c-statistic of 0.65 in the case of patients who underwent PCI.

Another study [28] assessed the role of CRUSADE, ACUITY, and HAS-BLED scores in predicting bleeding events associated with DAT for up to 24 months. The authors noted a similar discrimination power for CRUSADE and ACUITY risk scores (c-statistic 0.71 and 0.68 respectively), which was lower for the HAS-BLED score (c-statistic 0.63). However, a small number of bleeding events (53, 2.7%) were observed during follow-up, limiting the study results. 

Sharma et al. [27], developed and internally validated an original bleeding risk score for patients with PCI and subsequent DAT. The score achieved a modest performance, with a c-statistic of 0.67. Results are limited by the definition of bleeding used, BARC type ≥ 1, along with the exclusion of BARC type 5. 

Two studies [16,17], which evaluated the performance of bleeding risk scores in predicting in-hospital hemorrhagic events, showed concordant results, with a c-statistic value of 0.77 and 0.80, respectively, for the CRUSADE score. In addition, the ACTION risk score’s predictive performance was similar in both studies (c-statistic 0.78 and 0.75, respectively) but was slightly lower in the case of ACUITY-HORIZONS score (c-statistic 0.70). The clinical setting is essential, as both studies included patients with STEMI who underwent primary PCI.

Risk of bias and concern regarding applicability were assessed using the PROBAST checklist [13] designed for clinical models’ development and validation studies, and results are reported in Table S3. Overall, there was a high risk of bias, given that most studies are observational and retrospective. Most studies had low concern regarding applicability, as patients, predictors, and outcomes correspond to criteria of selection and inclusion in the present systematic review.

## 4. Discussion

Once DAT became a mandatory therapeutic regimen following PCI, the rate of ischemic events was significantly reduced at the cost of increased incidence of bleeding events. ACC/AHA, ESC, and EACTS recommend a personalized approach and careful evaluation of each patient’s ischemic and bleeding risks. Tailoring duration of DAT is one of the most critical concerns, as the bleeding risk can exceed the ischemic risk in certain circumstances. For this purpose, the DAPT risk scores are endorsed by ACC/AHA, ESC, and EACTS, which recommend the clinical use of both PRECISE-DAPT and DAPT scores. 

However, with the advent of more potent P2Y12 inhibitors and new generations of stents, current research is geared toward developing new risk scores or updating the validity of existing scores in the present-day clinical settings. 

The PRECISE-DAPT score was developed and validated in large cohorts of patients [22] who underwent PCI with stent implantation. This score helps adjust the duration of DAT (short versus standard/long). Nevertheless, Clopidogrel was the most used P2Y12 antagonist (88%) in the study as part of DAT, thus raising concern regarding applicability in the context of more potent P2Y12 inhibitors, which are widely prescribed. In addition, patients requiring triple antithrombotic medication were excluded from the study. Choi et al. [29] validated the PRECISE-DAPT score in a cohort of patients, which is somewhat closer to contemporary patients (almost all received DES, but more potent P2Y12 were prescribed to only 22% of non-HBR patients). Moreover, Gragnano et al. [31] validated both classic PRECISE-DAPT score and the four-item PRECISE-DAPT score (recently developed) in all-comer patients receiving DES reported a similar prediction power.

The DAPT score combines ischemic and bleeding risks, and American and European guidelines recommend tailoring the duration of DAT after 12 months of treatment (up to 30 months in long-term DAT). The derivation and validation cohorts [23] included only patients who had not experienced ischemic or hemorrhagic events during the first year, but those with long-term anticoagulant therapy were excluded. Clopidogrel or Prasugrel was administered as a P2Y12 antagonist. In addition, it also included patients who received either BMS or DES (89.3% of patients with bleeding events and 85.4% of patients without bleeding events). However, the DAPT score is not suitable for patients with a high risk of bleeding as a criterion for selecting BMS. That is why the DAPT score is addressed to carefully selected patients, comparable to those included in the derivation and validation studies. 

The PARIS bleeding risk score [24] was developed and validated in cohorts of patients who underwent PCI with DES from Europe and the USA, but its recommendation by guidelines in stratifying bleeding risk is unclear. The inclusion of patients treated with triple antithrombotic therapy at discharge is a possible advantage of this study. The PARIS risk score is appropriate for a limited group of patients, as those treated with BMS were excluded, and Clopidogrel was the P2Y12 antagonist used in the majority of cases. Unsurprisingly, long-term oral anticoagulation was associated with an increased incidence of hemorrhagic events and was included in the final prediction model, in contrast to the DAPT study, which does not cover this clinical area. In addition, the PARIS study provided a score predicting the risk of ischemic events, which could improve along with the bleeding risk score the selection of patients in whom extending the duration of DAT after one year is useful and less harmful. 

Abu-Assi et al. [15] attempted to compare the predictive performance of PARIS and PRECISE-DAPT bleeding risk scores in a cohort of patients with ACS and PCI from Spain. The particularities of this study are represented by a more significant proportion of patients treated with triple antithrombotic therapy at discharge (8.2% in comparison to PARIS derivation cohort—4.8% and PRECISE-DAPT study—0%) and a greater extent of Ticagrelor prescribed (22.9% versus PRECISE-DAPT cohort—3.9%). In addition, patients treated with DES, as well as those treated with BMS, were included. Both scores had a modest performance for predicting one-year hemorrhagic events, with a similar c-statistic value (0.73).

PRECISE-DAPT and PARIS bleeding risk scores were externally validated in another study by Bianco et al. [25] in patients with ACS and PCI with stenting. However, the cohort was different. As part of DAT, Aspirin in addition to Ticagrelor (61%) or Prasugrel (39%) were prescribed, thus excluding Clopidogrel from the therapeutic regimen. In addition, the proportion of patients treated with DES was greater (93%) than the proportion of patients treated with BMS (7%), which was somewhat different in comparison to PARIS derivation and validation cohorts, when only DES were used. This study’s population is closer to the contemporary patients in the context of availability of more potent P2Y12 inhibitors, slightly favoring PRECISE-DAPT score in stratifying bleeding risk. 

Ueki et al. [18] validated a new risk score, ARC-HBR, which was designed for patients who underwent PCI with stent implantation. Potent P2Y12 inhibitors (Ticagrelor or Prasugrel) were prescribed more frequently in the case of patients without a high risk of bleeding (52.5%) than in those with a high risk of hemorrhagic events (24.7%). On the one hand, the ARC-HBR score had a modest discriminatory power for bleeding events (c-statistic 0.69), with good sensitivity (68.5%) and negative predictive value (98.1%); on the other hand, specificity was lower (61.7%) with a poor positive predictive value (6.4%). ARC-HBR score was also validated by Cao et al. [19], in a contemporary real-world cohort of patients (almost all received DES), including those requiring anticoagulant drugs.

Raposeiras-Roubin et al. [26] developed and internally validated BleeMACS scores in a large cohort of patients in ACS with PCI and subsequent DAT involving ten different North American countries, South America, Europe, and Asia. The BleeMACS score was also externally validated in the SWEDE-HEART registry, including 96,239 consecutive patients with ACS. The discrimination power for bleeding events was slightly lower in the SWEDE-HEART cohort (c-statistic 0.65 for patients with PCI and 0.63 in those without PCI) than in the derivation cohort. Furthermore, the BleeMACS score’s performance was tested across different types of DAT in the external validation cohort. Hemorrhagic events’ discrimination power was similar for Aspirin plus Clopidogrel and Aspirin plus Ticagrelor (c-statistic 0.65).

Moreover, the role of BleeMACS score in stratifying bleeding events was evaluated in patients treated with different anticoagulant regimens in the SWEDE-HEART registry. The discriminatory power of BleeMACS score decreased in patients treated with triple antithrombotic therapy (c-statistic 0.60) but was slightly higher in patients treated with dual antithrombotic therapy, with oral anticoagulant and an antiplatelet agent (c-statistic 0.69). The study’s evident strength is represented by the large number of patients from different countries included in derivation and validation cohorts. In addition, the fact that the performance of the BleeMACS score in predicting bleeding events was evaluated in different antithrombotic therapies represents a clear advantage.

The CRUSADE risk score was initially developed and validated for predicting in-hospital bleeding events in patients with NSTEMI [33], ACUITY-HORIZONS, and ACTION scores—in patients with both STEMI and NSTEMI [34,35]. All three bleeding risk scores were evaluated by Flores-Rios et al. [16] for predicting in-hospital bleedings in patients with STEMI and primary PCI from a single center in Spain. All patients in the cohort received a loading dose of Clopidogrel and Aspirin followed by a maintenance dose, limiting current therapeutic regimens’ applicability with more potent P2Y12 antagonists. The authors also advocated for intravenous treatment with glycoprotein IIb-IIIa inhibitors (Abciximab), which is a strategy that could modify the bleeding risk profile. Considering all these factors in interpreting the results, CRUSADE and ACTION scores performed better in predicting in-hospital bleeding events than ACUITY-HORIZONS score.

The CRUSADE score was validated by Ariza-Sole et al. [17] in patients with STEMI who underwent primary PCI. Patients with chronic oral anticoagulation were excluded, and none of the participants received novel P2Y12 inhibitors. According to CRUSADE criteria, when hemorrhagic events were defined, the CRUSADE score had a better discriminatory power in predicting bleeding events than ACTION and Mehran scores, which is a difference that did not maintain when BARC criteria defined hemorrhagic events. The authors also observed that the CRUSADE score had a higher c-statistic value (0.86, 95% CI 0.77–0.96) in patients treated with glycoprotein IIb-IIIa inhibitors, and patients had a higher rate of major in-hospital bleeding events. Moreover, the CRUSADE score’s predictive power was similar in patients who underwent angiography using both the transradial or transfemoral approach. However, a low incidence of bleeding events during follow-up (33 patients, 3.1%) could be a source of bias in interpreting the results.

Costa et al. [28] evaluated the value of three risk scores, CRUSADE, ACUITY, and HAS-BLED, to predict hemorrhagic events in patients who underwent PCI with stenting, which was treated with short-term (6 months) or long-term (24 months) DAT. This is a non-classic indication for CRUSADE and ACUITY scores, as both were developed and validated for the prediction of in-hospital hemorrhagic events, not for out of hospital bleedings. The HAS-BLED score is also approved for stratifying bleeding risk in patients who need anticoagulant therapy, not in those with DAT. Nevertheless, CRUSADE and ACUITY scores had a better predictive performance for bleeding events than HAS-BLED. However, the DAT regimen consisted of Aspirin plus Clopidogrel, and none of the patients received novel P2Y12 inhibitors. The results are limited by fewer hemorrhagic events reported (53 patients, 2.7%).

Choi et al. [14] evaluated the predictive power of PRECISE-DAPT, ACUITY, and CRUSADE bleeding risk scores in a cohort of patients from South Korea who underwent PCI with stent implantation and subsequently DAT. Patients treated with oral anticoagulants and those with severe chronic renal failure were excluded. In addition, Clopidogrel was prescribed with predilection (94.9%), instead of Ticagrelor (0.8%) and Prasugrel (4.3%). Although ACUITY and CRUSADE risk scores were not developed to predict long-term bleeding events, the discriminatory power was similar to that reported for PRECISE-DAPT score.

Although American and European guidelines recommend only DAPT and PRECISE-DAPT scores, both have limitations because of differences between the cohort of patients, type of DAT, type of stent used in studies, and real-world patients. Given that there is a trend of high individualization in bleeding risk stratification in the case of patients treated with DAT, alternative risk scores validated in large cohorts of patients could be applied in a particular clinical scenario that is the closest to that analyzed in studies. Furthermore, a concern is raised about the applicability of bleeding risk scores in elderly patients. In this regard, Pavasini et al. [36] reported a similar efficiency of PRECISE-DAPT, PARIS, and BleeMACS risk scores, suggesting that these scores could also be used in older adults.

That is why all available data from patients should be carefully analyzed before using a prediction score, without neglecting the clinical judgment. None of the scores has a perfect prediction performance. Moreover, a novel trend is represented by the shortening of DAT duration [37] followed by treatment with a single antiplatelet agent reported by studies, with the same ischemic risk but with an improved hemorrhagic risk [38,39,40,41], which may change the bleeding profile of the patients in the future.

## 5. Conclusions

The existence of so many bleeding risk scores denotes the imperious need for a very accurate clinical model that could help adjust the type and duration of DAT to reduce the ischemic risk efficiently without increasing the bleeding risk. Each score has its benefits and limits derived from the characteristics of the development and validation cohorts and could be applied to a specific patient, in a particular clinical context, and at a specific time concerning the moment of PCI. Promising alternatives to PRECISE-DAPT scores are represented by ARC-HBR and BleeMACS scores, which are validated in large cohorts of patients. More prospective studies are needed in order to establish the relationship between clinical outcomes and the use of such clinical tools.

## Figures and Tables

**Table 1:** General characteristics of studies included in present systematic review.

Study	Type of Study	Risk Score	Enrollment (Time, Place)	Number of Patients	Clinical Setting	Age Median/Mean	Bleeding Events		
							Number (%)	Definition	Time
Costa et al., 2017 [22]	RTS	PRECISE-DAPT (derivation cohort)	139 different clinical sites from 12 countries worldwide	14,963	PCI + stent	65.0	218 (1.5)	TIMI major or minor bleeding	1 year
		PRECISE-DAPT (validation cohort—PLATO)	–	8595		61.0	145 (1.7)	TIMI major or minor bleeding	1 year
		PRECISE-DAPT (validation cohort—BernPCI)	Switzerland, between 23 February 2009, and 31 December 2014	6172		67.2	94 (1.5)	TIMI major or minor bleeding	1 year
Choi et al., 2018 [14]	RTS	PRECISE-DAPT ACUITY CRUSADE	Korea, between November 2008 and November 2015	904	PCI + stent	65.5	119 (13.2)	TIMI major or minor bleeding	1 year
							80 (8.8)	GUSTO moderate or severe	
							154 (17)	BARC ≥ 3a	
Abu-Assi et al., 2018 [15]	RTS	PRECISE-DAPT PARIS	Spain, between January 2012 and March 2015	1926	ACS + PCI + stent	65.1	136 (7.1)	BARC type 2, 3 or 5	1 year
							53 (2.8)	BARC type 3 or 5	
Ueki et al., 2020 [18]	OBS	ARC-HBR	Switzerland, between January 2009 and December 2016	12,121	PCI + stent	75.5 HBR	304 (6.4) HBR	BARC type 3 or 5	1 year
						62.8 non-HBR	140 (1.9) non-HBR		
Yeh et al., 2016 [23]	RTS	DAPT (derivation cohort)	11 countries, from August 2009 to May 2014	11,648	PCI + stent	61.3	215 (1.8)	GUSTO moderate or severe	12–30 m
		DAPT (validation cohort—PROTECT)	36 countries, from June 2007 through July 2014	8136		62.0	37 (0.5)		
Baber et al., 2016 [24]	OBS	PARIS (derivation cohort)	United States and Europe between July 2009 and December 2010	4190	PCI + drug eluting stent	67.8 MB	133 (3.3)	BARC type 3 or 5	2 year
						63.6 no MB			
		PARIS (validation cohort)	–	8130		63.6	296 (3.6)	Bleeding requiring hospitalization or transfusion	
Bianco et al., 2019 [25]	RTS	PRECISE-DAPT PARIS	12 European centers from January 2012 to December 2016 (RENAMI registry)	4424	ACS + PCI + stent	60.9	83 (1.88)	BARC type 3 or 5	14 m
Raposeiras-Roubin et al., 2018 [26,32]	RTS	BleeMACS (derivation + internal validation cohort)	15 hospitals from North and South America, Europe and Asia from November 2003 through June 2014	15,401	ACS + PCI	63.6	489 (3.2)	Any intracranial bleeding or any other bleeding leading to hospitalization and/or red blood transfusion ≥ 1 unit not related to procedures/surgery	1 year
Sharma et al., 2017 [27]	OBS	Original	10 hospitals from United States between 2009 and 2011 (PRISM study)	3128	PCI	64.5 (BARC ≥ 1)	2554 (81.6)	BARC type ≥ 1 and BARC type ≥ 2 (except BARC type 4 and 5)	1 year
Flores-Rios et al., 2012 [16]	OBS	CRUSADE ACUITY-HORIZONS ACTION	Spain, single hospital, between January 2006 and December 2010	1391	STEMI + primary PCI	64.0	136 (9.8)	Composite of intracranial or intraocular bleeding; access site hemorrhage requiring intervention; reduction in hemoglobin ≥ 4g/dL without or ≥ 3g/dL with overt bleeding source; reoperation for bleeding; blood transfusion	in-hospital
Ariza-Sole et al., 2013 [17]	OBS	CRUSADE ACTION Mehran	Spain, single hospital, between October 2009 and April 2012	1064	STEMI + primary PCI	61.7	33 (3.1)	Major bleeding defined by TIMI, BARC type 3 or 5	in-hospital
Costa et al., 2015 [28]	RTS	CRUSADE ACUITY HAS-BLED	Three Italian centers (PRODIGY study)	1946	PCI + stent	76.3 MB	53 (2.7)	Major bleeding defined by TIMI, GUSTO and BARC type 3 or 5	6 m/24 m
						68.9 no MB			
						62.8 non-HBR	140 (1.9) non-HBR		
Choi et al., 2020 [29]	Analy-sis from RCT	PRECISE-DAPT	31 centers in Republic of Korea (SMART-DATE trial)	2712	ACS + PCI	74.0 HBR	10 (1.5) HBR	BARC type 3–5	18 m
						57.7 non-HBR	6 (0.3) non-HBR		
Kawashima et al., 2020 [30]	Analy-sis from RCT	PRECISE-DAPT CRUSADE ACUITY	130 hospitals in 18 countries between July 2013 to November 2015	14,709	PCI	64.6	–	BARC type 3 or 5	30 d
Cao et al., 2020 [19]	OBS	ARC-HBR	Tertiary care center in New York, between January 2014 and December 2017	9623	PCI + stent	71.7 HBR	390 (9.1) HBR	Bleeding event requiring either hospitalization or blood transfusion	1 year
						61.8 non-HBR	172 (3.2) non-HBR		
Song et al., 2018 [20]	OBS	DAPT PARIS	Single hospital in China, from 1 January to 31 December 2013	6088	PCI + DES	58.3	30 (0.50)	BARC type 3 or 5	2 year
Ueda et al., 2018 [21]	OBS	DAPT	Sweden, between 1 January 2006 and 31 December 2013	41,101	PCI + stent	61.2 high DAPT score	311 (0.75)	GUSTO moderate or severe	30 m
Gragnano et al., 2020 [31]	Analy-sis from RCT	PRECISE-DAPT (GLOBAL LEADERS trial)	130 sites in 18 countries	14,928	PCI + DES	62.5 low PRECISE-DAPT	163 (2.18) control group	BARC type 3 or 5	1 year and 2 year
						75.0 high PRECISE-DAPT			
		PRECISE-DAPT (GLASSY trial)	Sub-study of GLOBAL LEADERS trial with patients enrolled at the 20 highest recruiting sites	7134		62.7 low PRECISE-DAPT	91 (2.54) control group		
						75.9 high PRECISE-DAPT			

RTS = retrospective; OBS = observational; RCT = randomized controlled trial; HBR = high bleeding risk; MB = major bleeding.

## Supplementary Materials

The following are available online at https://www.mdpi.com/2227-9032/9/2/148/s1, Table S1: Definitions of bleeding risk scores, Table S2: Comparative discrimination power of bleeding risk scores reported in clinical studies, Table S3: Risk of bias and applicability assessment based on PROBAST checklist.
