# Supplementary material for: A Systematic Review on Bleeding Risk Scores’ Accuracy after Percutaneous Coronary Interventions in Acute and Elective Settings
Source: Healthcare (Basel). 2021 Feb 2;9(2):148. doi: 10.3390/healthcare9020148 (PMC7912805; doi:10.3390/healthcare9020148)
Supplement: Supplementary file 1 [file healthcare-09-00148-s001.pdf]

healthcare MDPI

Table S1. Definitions of bleeding risk scores.

Definitions of bleeding risk scores		
**PRECISE-DAPT score**		
• Hemoglobin<br>• White blood cell count<br>• Age<br>• Creatinine clearance<br>• Prior bleeding		Score ≥ 25 – Short DAT<br>Score < 25 – Standard or long DAT
**DAPT score (after 12 months of uneventful DAT)**		
• Age ≥ 75 (-2p)<br>65 to < 75 (-1p)<br>< 65 (0p)<br>• Cigarette smoking (+1p)<br>• Diabetes mellitus (+1p)<br>• MI at presentation (+1p)	• Prior PCI or prior MI (+1p)<br>• Paclitaxel-eluting stent (+1p)<br>• Stent diameter < 3 mm (+1p)<br>• CHF or LVEF < 30% (+2p)<br>• Vein graft stent (+2p)	Score ≥ 2 – Long DAT<br>Score < 2 – Standard DAT
**ARC-HBR criteria**		
Major risk factors:<br>• Anticipated use of long-term oral anticoagulation<br>• Severe or end-stage CKD (eGFR < 30 mL/min)<br>• Hemoglobin < 11 g/dL<br>• Spontaneous non-intracranial bleeding requiring hospitalization or transfusion in the past 6 months or at any time, if recurrent<br>• Moderate or severe baseline thrombocytopenia (< 100 x 10^9/L)<br>• Chronic bleeding diathesis<br>• Liver cirrhosis with portal hypertension<br>• Active malignancy (excluding non-melanoma skin cancer) within the past 12 months<br>• Previous spontaneous intracranial hemorrhage (at any time)<br>• Previous traumatic intracranial hemorrhage within the past 12 months<br>• Presence of brain arteriovenous malformation<br>• Moderate or severe ischemic stroke within 6 months<br>• Planned non-deferrable non-cardiac major surgery on DAT<br>• Recent major surgery or major trauma within 30 days before PCI	Minor risk factors:<br>• Age ≥ 75<br>• Moderate CKD (eGFR 30-59 mL/min)<br>• Hemoglobin 11-12.9 g/dL for men and 11-11.9 g/dL for women<br>• Spontaneous non-intracranial bleeding requiring hospitalization or transfusion within the past 12 months not meeting the major criterion<br>• Long-term use of oral NSAIDs or steroids<br>• Any ischemic stroke at any time not meeting the major criterion	High bleeding risk = at least 1 major criterion or 2 minor criteria
**PARIS bleeding risk score**		
• Age < 50 (0p)<br>50-59 (+1p)<br>60-69 (+2p)<br>70-79 (+3p)<br>≥ 80 (+4p)<br>• Current smoking (+2p)	• BMI, kg/m^2 < 25 (+2p)<br>25-34.9 (0p)<br>≥ 35 (+2p)<br>• Anemia (+3p)<br>• Creatinine clearance < 60 mL/min (+2p)<br>• Triple therapy on discharge (+2p)	Low bleeding risk = 0 – 3p<br>Intermediate bleeding risk = 4 – 7p<br>High bleeding risk ≥ 8p

Table S2. Comparative discrimination power of bleeding risk scores reported in clinical studies.

Study	Risk scores evaluated	Results	
		c-statistic/AUROC	Confidence interval (CI)
**Costa el al, 2017**	PRECISE DAPT (derivation cohort)	0.73	95% CI (0.61 – 0.85)
	PRECISE DAPT (validation cohort – PLATO trial)	0.70	95% CI (0.65 – 0.74)
	PRECISE DAPT (validation cohort – BernPCI)	0.66	95% CI (0.61 – 0.71)
**Choi et al, 2018**	PRECISE DAPT	0.81 (BARC)	95% CI (0.78 – 0.84)
		0.75 (TIMI)	95% CI (0.72 – 0.78)
		0.82 (GUSTO)	95% CI (0.80 – 0.85)
	ACUITY	0.81 (BARC)	95% CI (0.78 – 0.83)
		0.75 (TIMI)	95% CI (0.72 – 0.78)
		0.79 (GUSTO)	95% CI (0.76 – 0.82)
	CRUSADE	0.79 (BARC)	95% CI (0.76 – 0.81)
		0.73 (TIMI)	95% CI (0.70 – 0.76)
		0.81 (GUSTO)	95% CI (0.78 – 0.83)
**Abu-Assi et al, 2018**	PRECISE DAPT	0.73	95% CI (0.67 – 0.79)
	PARIS	0.73	95% CI (0.66 – 0.80)
**Ueki et al, 2020**	ARC-HBR	0.67	95% CI (0.64 – 0.70)
	PRECISE-DAPT	0.67	95% CI (0.65 – 0.70)
	PARIS	0.68	95% CI (0.65 – 0.71)
**Yeh et al, 2016**	DAPT (derivation cohort)	0.68	95% CI (0.65 – 0.72)
	DAPT (validation cohort – PROTECT trial)	0.64	95% CI (0.55 – 0.73)
**Baber et al, 2016**	PARIS (derivation cohort)	0.75	–
	PARIS (validation cohort – ADAPT-DES)	0.64	–
**Bianco et al, 2019**	PRECISE DAPT	0.653	95% CI (0.59 – 0.71)
	PARIS	0.593	95% CI (0.52 – 0.65)
**Raposeiras-Roubin et al, 2018**	BleeMACS (derivation cohort)	0.71	95% CI (0.68 – 0.74)
	BleeMACS (internal validation co-hort)	0.72	95% CI (0.67 – 0.76)
**Sharma et al, 2017**	Original	0.67	–
**Flores-Rios et al, 2012**	CRUSADE	0.77	95% CI (0.75 – 0.79)
	ACUITY-HORIZONS	0.70	95% CI (0.67 – 0.72)
	ACTION	0.78	95% CI (0.76 – 0.80)
**Ariza-Sole et al, 2013**	CRUSADE	0.80	95% CI (0.73 – 0.87)
	ACTION	0.75	95% CI (0.69 – 0.80)
	Mehran	0.76	95% CI (0.71 – 0.82)
**Costa et al, 2015**	CRUSADE	0.71	95% CI (0.64 – 0.77)
	ACUITY	0.68	95% CI (0.61 – 0.75)
	HAS-BLED	0.63	95% CI (0.56 – 0.70)
**Choi et al, 2020**	PRECISE-DAPT	0.754	95% CI (0.65 – 0.85)
**Kawashima et al, 2020**	PRECISE-DAPT	0.648	95% CI (0.61 – 0.67)
	CRUSADE	0.641	95% CI (0.60 – 0.67)
	ACUITY	0.633	95% CI (0.60 – 0.66)
**Cao et al, 2020**	ARC-HBR	0.68	95% CI (0.65 – 0.71)
**Song et al, 2018**	DAPT	0.71	–
	PARIS	0.56	–
**Ueda et al, 2018**	DAPT	0.49	95% CI (0.45 – 0.53)

Table S2. Comparative discrimination power of bleeding risk scores reported in clinical studies – continued.

Study	Risk scores evaluated	Results	
		c-statistic/AUROC	Confidence interval (CI)
**Gragnano et al, 2020**	PRECISE-DAPT (GLOBAL LEADERS) at 1 year	0.62	95% CI (0.58 – 0.67)
	4-item PRECISE-DAPT (GLOBAL LEADERS) at 1 year	0.60	95% CI (0.55 – 0.65)
	PRECISE-DAPT (GLOBAL LEADERS) at 2 years	0.63	95% CI (0.59 – 0.67)
	4-item PRECISE-DAPT (GLOBAL LEADERS) at 2 years	0.61	95% CI (0.57 – 0.65)
	PRECISE-DAPT (GLASSY) at 1 year	0.64	95% CI (0.58 – 0.71)
	4-item PRECISE-DAPT (GLASSY) at 1 year	0.63	95% CI (0.57 – 0.69)
	PRECISE-DAPT (GLASSY) at 2 years	0.66	95% CI (0.61 – 0.72)
	4-item PRECISE-DAPT (GLASSY) at 2 years	0.65	95% CI (0.60 – 0.71)

Table S3. Risk of bias and applicability assessment based on PROBAST checklist.

Study	ROB*				Applicability (AP)			Overall	
	PRT**	PRD***	Outcome	Analysis	PRT	PRD	Outcome	ROB	AP
Costa, 2017	+	+	+	-	+	+	+	-	+
Choi, 2018	+	?	?	-	+	+	+	-	+
Abu-Assi, 2018	+	?	?	-	+	+	+	-	+
Ueki, 2020	+	?	?	-	+	+	+	-	+
Yeh, 2016 (derivation)	+	?	?	?	+	+	+	?	+
Baber, 2016 (derivation)	+	?	?	?	+	+	+	?	+
Bianco, 2019	+	?	?	-	+	+	+	-	+
Raposeiras-Roubin, 2018 (derivation)	+	?	?	-	+	+	+	-	+
Sharma, 2017	+	?	-	?	+	+	-	-	-
Flores-Rios, 2012	+	?	?	?	+	+	+	?	+
Ariza-Sole, 2013	+	+	?	-	+	+	+	-	+
Costa, 2015	+	?	?	-	+	+	+	-	+
Ueki, 2020	+	?	?	-	+	+	+	-	+
Choi, 2020	+	+	+	-	+	+	+	-	+
Kawashima, 2020	+	+	?	?	+	+	+	?	+
Cao, 2020	+	?	?	-	+	+	+	-	+
Song, 2018	+	?	?	-	+	+	+	-	+
Ueda, 2018	+	?	?	-	+	+	+	-	+
Gragnano, 2020	+	+	+	-	+	+	+	-	+

ROB* = risk of bias; PRT** = participants; PRD*** = predictors;
+ indicates low ROB/low concern regarding applicability; - indicates high ROB/concern regarding applicability; ? indicates unclear ROB/unclear concern regarding applicability
